# Prevalence, causes and outcomes of war-related civilian injuries in Ethiopia’s war-torn Tigray region: a community-based descriptive study

**DOI:** 10.1186/s13104-023-06640-4

**Published:** 2023-11-27

**Authors:** Akeza Awealom Asgedom, Abenezer Etsedingl, Teklehaimanot Tekle Hailemariam, Mengistu Hagazi Tequare, Tesfay Hailu, Amanuel Tesfay Tsegay, Abraha Gebreegziabher Hailu, Simret Niguse Weldebirhan, Melaku Hailu, Negash Abreha Weldesenbet, Girmatsion Fisseha, Yibrah Alemayehu

**Affiliations:** 1 Department of Environmental Health Science, School of Public Health, College of Health Sciences, Mekelle University, Mekelle, Tigray, Ethiopia; 2 Emergency Medical Services Case Team, Tigray Health Bureau, Mekelle, Tigray, Ethiopia; 3 Department of Physiotherapy, School of Medicine, College of Health Sciences, Mekelle University, Mekelle, Tigray, Ethiopia; 4 Department of Health Systems, School of Public Health, College of Health Sciences, Mekelle University, Mekelle, Tigray, Ethiopia; 5 Department of Epidemiology, School of Public Health, College of Health Sciences, Mekelle University, Mekelle, Tigray, Ethiopia; 6 Department of Anatomy and Embryology, Division of Biomedical Sciences, School of Medicine, College of Health Sciences, Mekelle University, Mekelle, Tigray, Ethiopia; 7 Department of Pediatrics and Child Health, School of Medicine, College of Health Sciences, Mekelle University, Mekelle, Tigray, Ethiopia; 8 Department of Emergency and Critical Care Nursing, School of Nursing, College of Health Sciences, Mekelle University, Mekelle, Tigray, Ethiopia; 9 Department of Reproductive Health, School of Public Health, College of Health Sciences, Mekelle University, Mekelle, Tigray, Ethiopia; 10 Tigray Health Bureau, Mekelle, Tigray, Ethiopia

**Keywords:** Cause, Civilian Injury, Ethiopia, Tigray, Outcome, Prevalence, War

## Abstract

**Objective:**

War and armed conflicts are the major causes of mortality, morbidity and disability. This study was aimed at assessing the prevalence of injury, cause and its outcome among civilians during the war in Tigray, Northern Ethiopia.

**Results:**

A community based cross sectional study was conducted to collect data from a total of 4,381 sample households. Descriptive analysis was applied and the data are presented using frequencies, percentages, tables and statements. Of the study participants, 6.9% (95% CI: 6.2%, 7.6%) of civilians encountered any kind of war-related physical injury. About Two-third (95% CI: 59%, 73%) of the physical injuries were caused by bullet followed by heavy artillery shelling (proportion = 23%; 95% CI: 17%, 29%). Painfully, about 44% (95% CI: 37%, 50%) faced death following injuries and the other 56.2% (95% CI: 50%, 62.5%) either survived or encountered disability. Post war rehabilitation for the disabled is recommended to enable them live healthy, dignified, independent and productive citizens.

## Background

War and armed conflicts are a major causes of mortality, morbidity and disability. Unfortunately, civilians have been targeted of war-related conflict and increased in the last one century of all casualties, 5% in World War I, 50% in World War II, over 80% in the US war in Vietnam, and over 90% in 1986 [1]. In 2020 there were 1.8 billion people living in conflict affected areas globally [2, 3].

War and armed conflicts are significant contributors to disease burden, especially in low and middle income countries [4]. The burden of war related injury varies from country to country in developing nations. For instance, a study from Iraq showed a 10.7% prevalence of injury [5] while findings from Palestine revealed 0.64% [6] .

Africa pays more to conflict-related injuries and deaths than any other continent. Almost 24 sub-Saharan countries experienced armed conflicts in the period of 1960s to 2008 [7]. World Bank reported one in every three African countries were affected directly or indirectly by armed conflicts. This accounts 53.96% of death and 53.07% of Disability Adjusted Life Years (DALYs) due to war from the world [8, 9]. At the last decade of the 20th century, civilian deaths in Africa ranged from 25,000–1,000,000 in some African countries [4]. In previous decades, Africa had faced various consequences of conflicts in different countries [9–11] However, most of the studies show a heterogeneous and inconsistent presentation of findings [12].

In Ethiopia’s Tigray region, a full-blown drastic war erupted in November 2020 [13, 14]. Following this, Tigray has seen the worst human suffering ever [15, 16]. Adequate information should be generated for rehabilitation and evidence-based decision-making purposes on the burden of the war. However, there exists a huge gap in evidence on what has been happening to Tigray and what is empirically known and documented. On top of this, there is no documented evidence on prevalence, cause and outcome of armed conflict on civilians in Tigray. Hence, this community-based study was aimed at assessing the prevalence of injury, cause and its outcome among civilians during the war in Tigray from November 2020 to June 2021.

## Methods

### Study design and setting

A community-based study was conducted in six zones of Tigray, namely Southern, South Eastern, Mekelle, Eastern, Central and North Western Zones of Tigray. There are seven zones that make up the Tigray regional state. However, the study did not include Western Tigray as it was unreachable for security reasons. Tigray has 93 districts (locally known as Woredas) and the study considered 52 Woredas from the six zones. The study was conducted from August 4 to August 20, 2021.

### Study population and sampling technique

This study was part of a large-scale ‘Key Performance Indicators for Health survey (primarily examining for maternal health, child health, nutritional status, WASH, and civilian injuries) conducted in 52 randomly selected woredas (woreda is an equivalent term for district) of Tigray Regional State in northern Ethiopia. The study is based on the Ethiopian Demographic and Health Survey (EDHS), which serves as the sampling area. Accordingly, four enumeration areas (locally referred to as ‘kebeles’ or ‘tabias’) per selected woreda were considered for the study. In total, there were 208 enumeration areas from 52 woredas. Twenty households in each enumeration area were considered for data collection, resulting in a total sample of 4,160 households. However, additional data were collected in some enumeration areas, resulting in a total sample of 4,381 households that was used for the analysis. The study followed a multistage sampling procedure (zone, woreda, kebele/tabia and households) and each households were selected by lottery. The household heads of the households were the subjects of this study.

### Inclusion and exclusion criteria

The inclusion criteria for the study participants were households with children under one year of age in their household, and the others were excluded. The main purpose of including households with children under one year of age was to collect maternal and child health indicators as part of the large survey of key health care performance indicators.

### Questionnaire development and data collection

A pre-tested questionnaire were used to collect data from households and asked questions regarding presence of war related civilian injury, cause and the main outcomes of the injury. The questionnaire was developed for this purpose by reviewing previously published articles. Data was collected by trained health extension workers. A supervisor was assigned to follow and monitor the overall activities of the data collection process.

### Data quality control

The questionnaire used for data collection was translated from English to Tigrigna and then back translated from Tigrigna to English by another language translator for checking the consistency of the language. Pre-test of questionnaire was done out of the study site before the actual data collection. Training was given for data collectors on the purpose of the study, how to collect the data, and ethical principles. Questionnaire was also checked for completeness each day of the data collection by the supervisors.

#### Data Processing and Analysis

Collected data was entered in to Epi Data version 3.1 software and then transferred in to SPSS, version 25 software for analysis. Descriptive analysis was applied and the data were presented in frequencies, percentages, tables and statements.

#### Operational definitions

**Disability** was defined as a limitation to normal activities from the injury described during the war time at the time of data collection.

**Injury** was defined as any intentional or unintentional physical harm that required medical care, whether received or not, and with or without an intervention, and which resulted in loss or reduction in normal activities for a period of time (November 4, 2020 to June 2021). The frequencies of response only considered a yes/no answers and for yes response it only counted as having one weight and the unit of analysis was at individual level, not at household level.

**Prewar** was the time before November 4, 2020.

**During war** was a time from November 4, 2020 to June 2021.

**Household (HH)** was defined as a group of persons living together in a dwelling with a separate outer door. One household consists an average of 5 individuals.

**Tabia/Kebele** is the lowest administrative unit in a Woreda.

**Woreda** is the equivalent name for district.

## Results

### General information

A total of 4,381 households participated in this study. Nearly 7% (95% CI: 6.2%, 7.6%) encountered any kind of war-related civilian physical injury. About Two-third (95% CI: 59%, 73%) of the injuries were caused by bullet followed by heavy artillery shelling (proportion = 23%; 95% CI: 17%, 29%). Painfully, about 44% (95% CI: 37%, 50%) faced death following injuries (Table 1).

**Table 1:** Zonal and sex distribution of study participants and overall prevalence, causes and outcomes of war-related injuries in Tigray, Ethiopia (N = 4,381)

Characteristics	Frequency	Percent	95% Confidence Interval	
			Lower	Upper
**Zone (n = 4379)**				
South	533	12.2	11.2	13.2
South Eastern	479	10.9	10.0	11.8
Mekelle	297	6.8	6.1	7.5
Central	1155	26.4	25.1	27.7
Eastern	1224	28.0	26.6	29.3
North Western	691	15.8	14.7	16.9
**Sex of respondent**				
Female	3308	75.5	74.2	76.7
Male	1073	24.5	23.3	25.8
**Any war related injury (n = 4339)**				
No	4041	93.1	92.4	93.8
Yes	298	6.9	6.2	7.6
**Cause of injury (n = 181)**				
Bullet	120	66.3	59.1	72.9
Military explosive/land mine	14	7.7	3.9	12.2
Heavy artillery	41	22.7	17.1	28.7
Air bombardment	6	3.3	1.1	6.1
**Outcome of injury (n = 240)**				
Death	105	43.8	37.5	50.0
Physical disability	135	56.2	50.0	62.5

### Zonal distribution of war-related physical injury prevalence, causes and outcomes

Eastern, Central, Southern and Northwestern zones had high civilian injury prevalence, giving 9.3%, 8.5%, 6% and 6%, respectively. Bullet injury was the most common cause in Northwestern zone while heavy artillery in Eastern and air bombardment in Southeastern zones (Table 2).

**Table 2:** Zonal prevalence, cause and outcomes of war-related injuries in Tigray, Ethiopia (N = 4339)

Variable		Frequency and percentage (n, %)						
		**Total** **(n = 4,339)**	Southern (n = 533)	South eastern (n = 479)	Mekelle (n = 297)	Central (n = 1,122)	Eastern (n = 1217)	North western (n = 689)
Number of injury due to war		**298(6.9%)**	32(6%)	13(2.7%)	4(1.3%)	95(8.5%)	113(9.3%)	41(6%)
Cause of injury (n = 181)	Bullet/Gunshot	**120/181**	13/18	6/11	2/4	40/67	43/56	16/25
	Military explosive	**14/181**	0	0	0	4/67	6/56	2/25
	Heavy artillery	**41/181**	5/18	1/11	2/4	23/67	7/56	5/25
	Air bombardment	**6/181**	0	4/11	0	0	0	2/25
Outcome (n = 240)	Death	**105/240**	12/30	8/9	2/4	36/78	28/80	19/37
	Physical disability	**135/240**	18/30	1/9	2/4	42/78	52/80	18/37

N.B. For cause and outcome of injuries, percentage was not calculated, but the frequency and denominator of each variables are written

## Discussion

The overall result of the study showed a prevalence of 6.9% of war-related civilian injuries. Gunshot wounds were the most common cause of civilian physical injury. Injured study participants either survived or died.

The study found that during the first eight months of the war in Tigray, 13.8 out of every 1,000 residents suffered some form of war-related injury. Based on the population projection for Ethiopian fiscal year 2014 [17], the number of civilian injuries in the region is estimated to be about 80,000 out of 5.777 million Tigrayans. The prevalence of injuries in the present study is higher than in studies conducted in Palestinian- Gaze (6.4 per 1000 population [6], Baghdad, Iraq (4.6 to 5 per 1000 populations) [5, 18], Libya (7.1 per 1000 populations) [11].

This may be due to the nature of the full-blown war, which involved many internal and external actors in the Tigray War. The actors include the Eritrean National Defense Forces, Ethiopian National Defense Forces, Amhara Special Forces, Amhara Militia, and Amhara Fano Vigilante Group [15, 19] on one side and the Tigray forces on the other. In the present study, there were large differences in the prevalence of injuries in the different zones of the region, ranging from 1.3 to 9.3%. In the eastern, central, southern, and northwestern zones, the prevalence of civilian injuries was high at 9.3%, 8.5%, 6%, and 6%, respectively. In most areas, gunshot wounds were the most common cause. Heavy artillery was also a common cause of injury in the central zone, while air strikes were the most common cause in the southeastern zones.

The prevalence of deaths due to war-related civilian physical injuries was 44% in the present study. This result is consistent with findings from Iraq, where the mortality rate was 39.1% [5].

However, the current study does not agree with the results of a study from Libya, in which 20.1% of injury cases were classified as fatal [11]), which is lower than our result. The high prevalence of fatalities among the injured in the present study may be due to the collapsed health care system of Tigray [14, 20], which makes it difficult to access medications after injury. The final outcome of the 56% injured civilians is not precisely known. They may have either survived or been permanently disabled. However, such detail study is not well addressed in the present study and this could be addressed in the future.

In the current study, a high proportion of injuries were caused by bullets, which is consistent with the results from Iraq, where gunshots were the main cause of injury [5]. However, the present result contrasts with the results from Palestine and Iraq, where explosions and blasts were the most common causes of war injuries [5, 6]. Targeted civilian actions may have contributed to the higher number of gunshot wounds in this study. For example, the Ethiopian National Defense Army killed dozens of civilians in Debre Abay [21] and Mahbere Dego [22, 23] in central Tigray. Similarly, the Ethiopian National Defense Forces killed 170 civilians in Bora [24] in South Tigray, which may justify the targeted violations of civilians.

## Conclusions

The results of this study suggest a higher prevalence of war-related injuries among civilians. Bullets were the leading cause of injury, followed by heavy artillery. Nearly half of the injured civilians were dead. The results of this study could help in the post-war rehabilitation of disabled civilians so that they can lead healthy, dignified, independent, and productive lives.

### Limitations

The study has some limitations. First, it was part of a larger survey of key health performance indicators that covered limited scope and depth related to injuries. It answers only a few questions about the prevalence and causes of injuries, while the injury outcomes are limited in their depth of classification. The inclusion criteria used in the larger survey exclude households with children older than one year, which could change the prevalence rate of civilian injuries. Based on the results, we recommend a comprehensive study for the western parts of Tigray and the inclusion of households with children older than 1 year, which were not included in the present study. In addition, socioeconomic variables, type and severity of injuries need to be considered in future studies. In addition, it is recommended that future studies identify the functional status and type of disability of injured civilians.

## Data Availability

The datasets used and/or analysed during the current study are available from the corresponding author on reasonable request.

## Abbreviations

BBC: British Broadcast Corporation
CI: Confidence Interval
CNN: Cable News Network
DALYs: Disability Adjusted Life Years
EDHS: Ethiopian Demographic Health Survey
HH: Household
MU-IRB: Mekelle University Institutional Review Board
Ref.No.: Reference Number
SPSS: Statistical Package for Social Science
